# Supplementary figures and images for: Essential role of hyperacetylated microtubules in innate immunity escape orchestrated by the EBV-encoded BHRF1 protein
Source: PLoS Pathog. 2022 Mar 11;18(3):e1010371. doi: 10.1371/journal.ppat.1010371 (PMC8942261; doi:10.1371/journal.ppat.1010371)

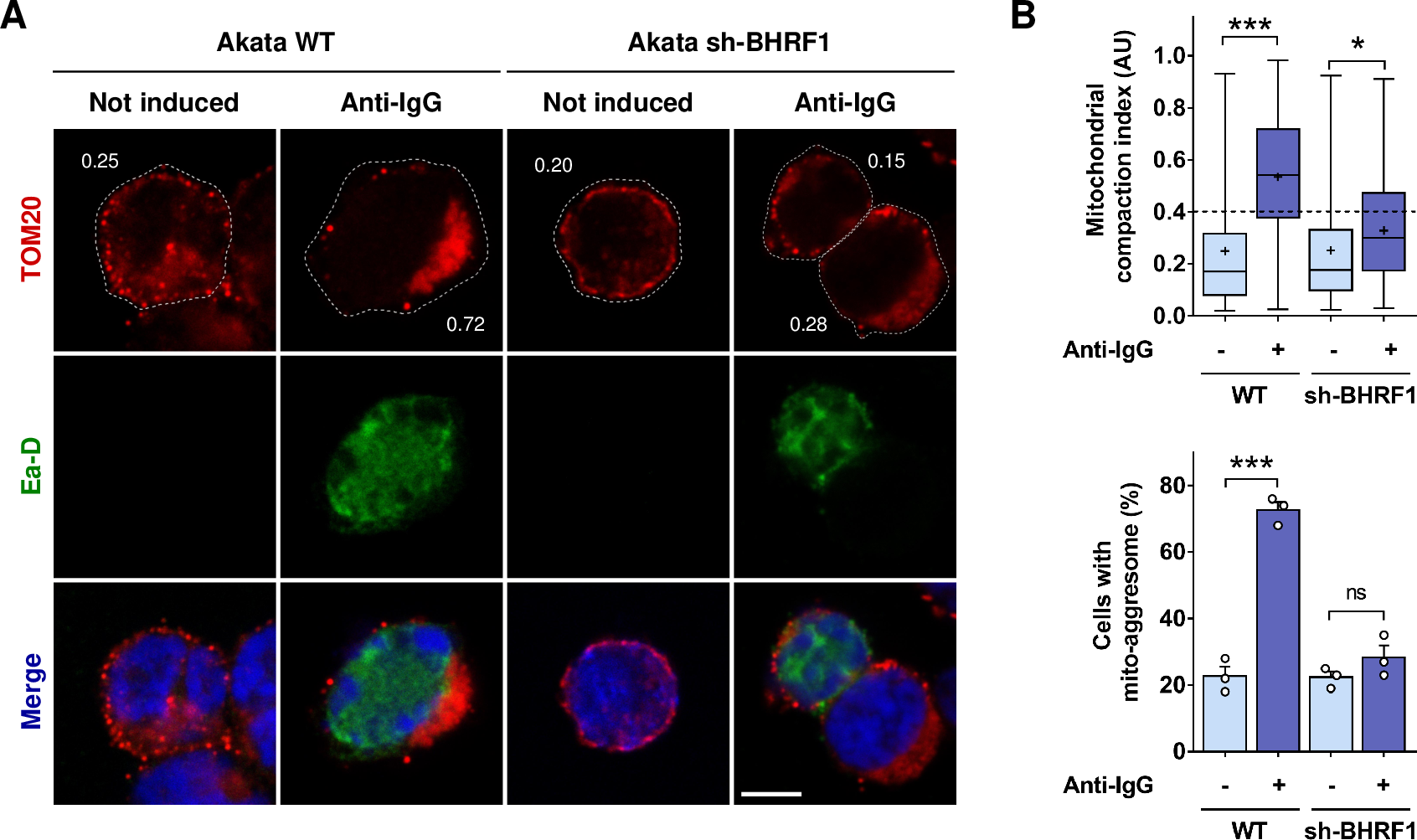

Supplement: S1 Fig — (A-B) Akata WT and sh-BHRF1 cells were treated or not with anti-human IgG for 24 h and then fixed. (A) Confocal images of cells immunostained for TOM20 and Ea-D. Nuclei were stained with DAPI. Values of mitochondrial CI are indicated. Scale bars: 5 μm. (B) Quantification of CI and percentage of cells with a mito-aggresome (n = 20 cells per condition). Data represent the mean ± SEM of three independent experiments. ns = non-significant; * P < 0.05; *** P < 0.001 (Student’s t-test). (TIF) [file ppat.1010371.s001.tif]

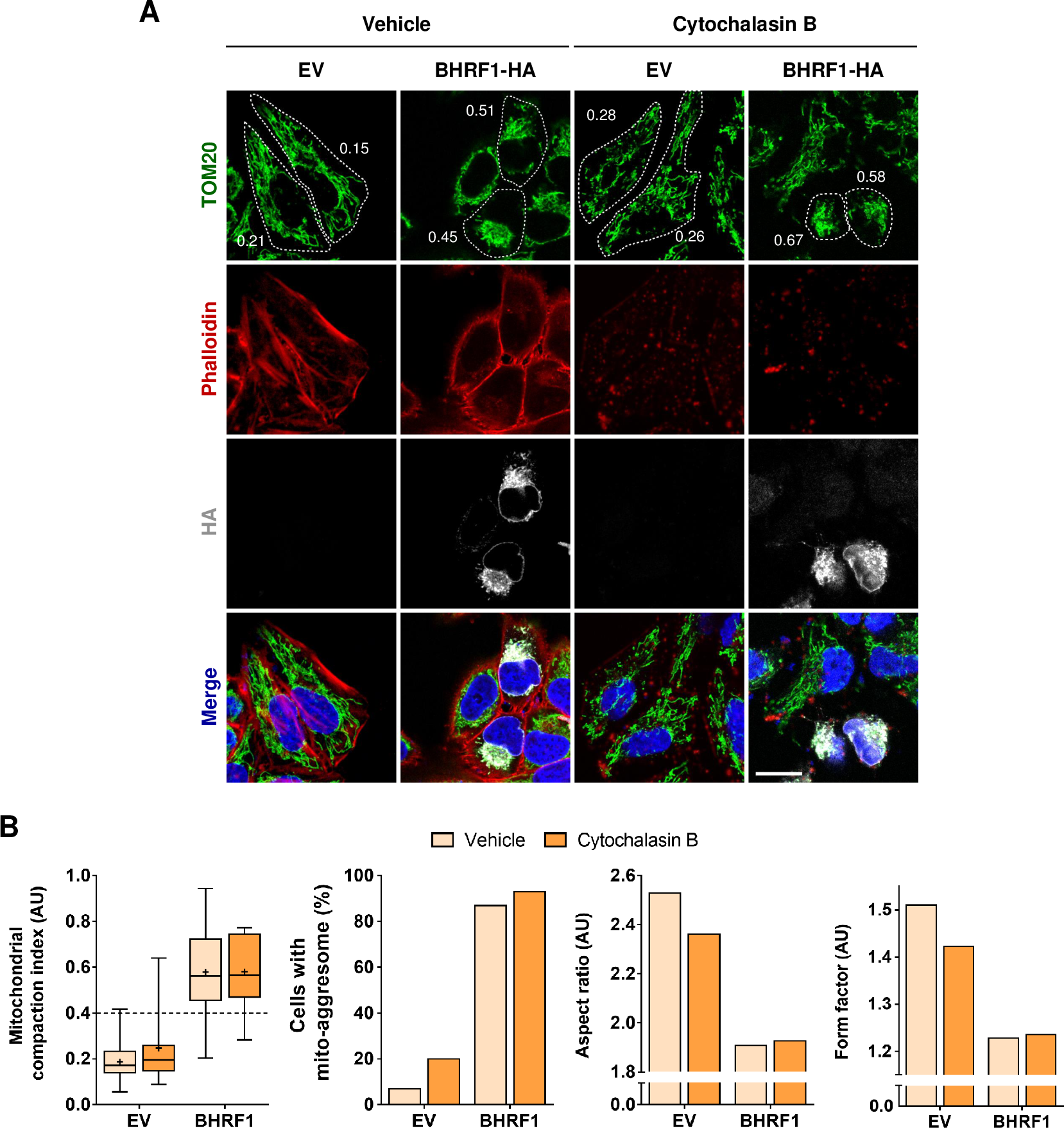

Supplement: S2 Fig — (A-B) HeLa cells were transfected with BHRF1-HA plasmid (or EV) for 24 h. For the final 30 min, cells were treated with cytochalasin B to disrupt the F-actin network and then were fixed. (A) Confocal images. Cells were immunostained for TOM20 and HA. The F-actin network was labeled with phalloidin and the efficiency of the treatment with cytochalasin B was confirmed by the loss of F-actin staining. Nuclei were stained with DAPI. Values of mitochondrial CI are indicated on representative cells. Scale bar: 20 μm. (B) Quantification of CI, percentage of cells with a mito-aggresome and mitochondrial fission parameters (n = 20 cells per condition). (TIF) [file ppat.1010371.s002.tif]

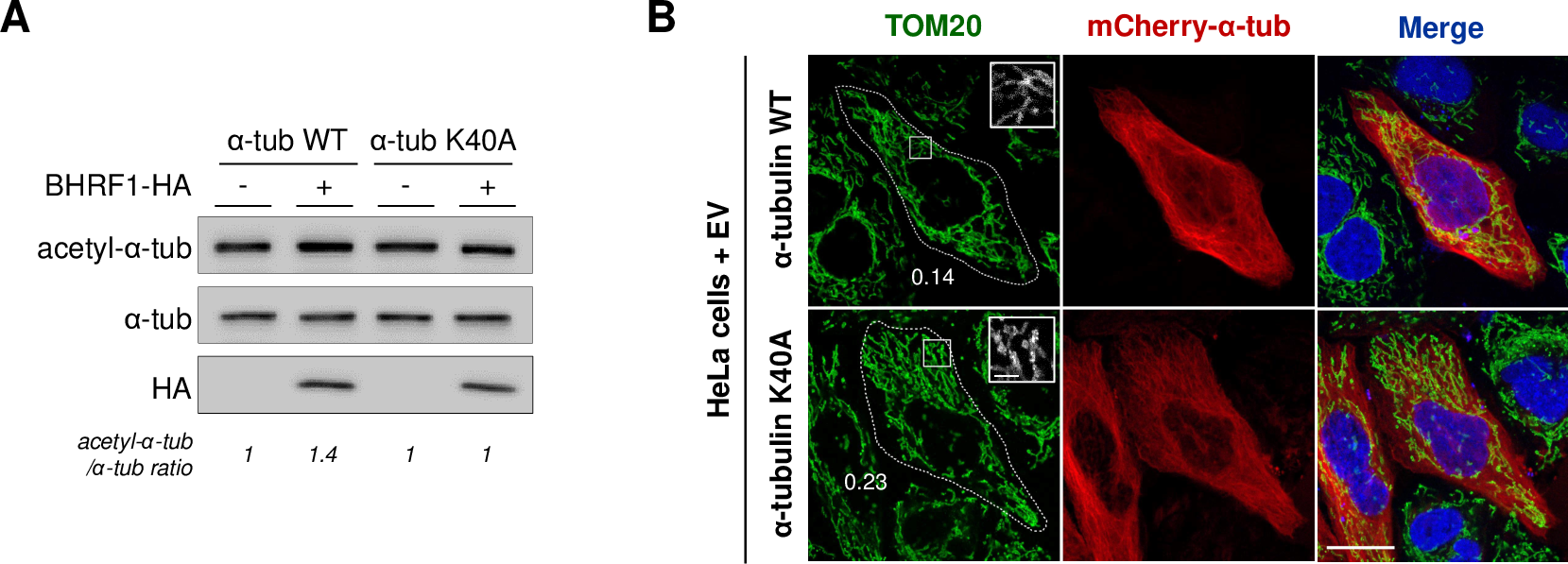

Supplement: S3 Fig — HeLa cells were co-transfected with plasmids encoding BHRF1-HA (or EV) and mCherry-α-tubulin K40A (or WT). (A) Immunoblot analysis of acetyl-α-tubulin, α-tubulin and HA. (B) Confocal images of EVtransfected cells immunostained for TOM20. Nuclei were stained with DAPI. Scale bars: 10 μm and 4 μm for insets. Values of mitochondrial CI are indicated on representative cells. (TIF) [file ppat.1010371.s003.tif]

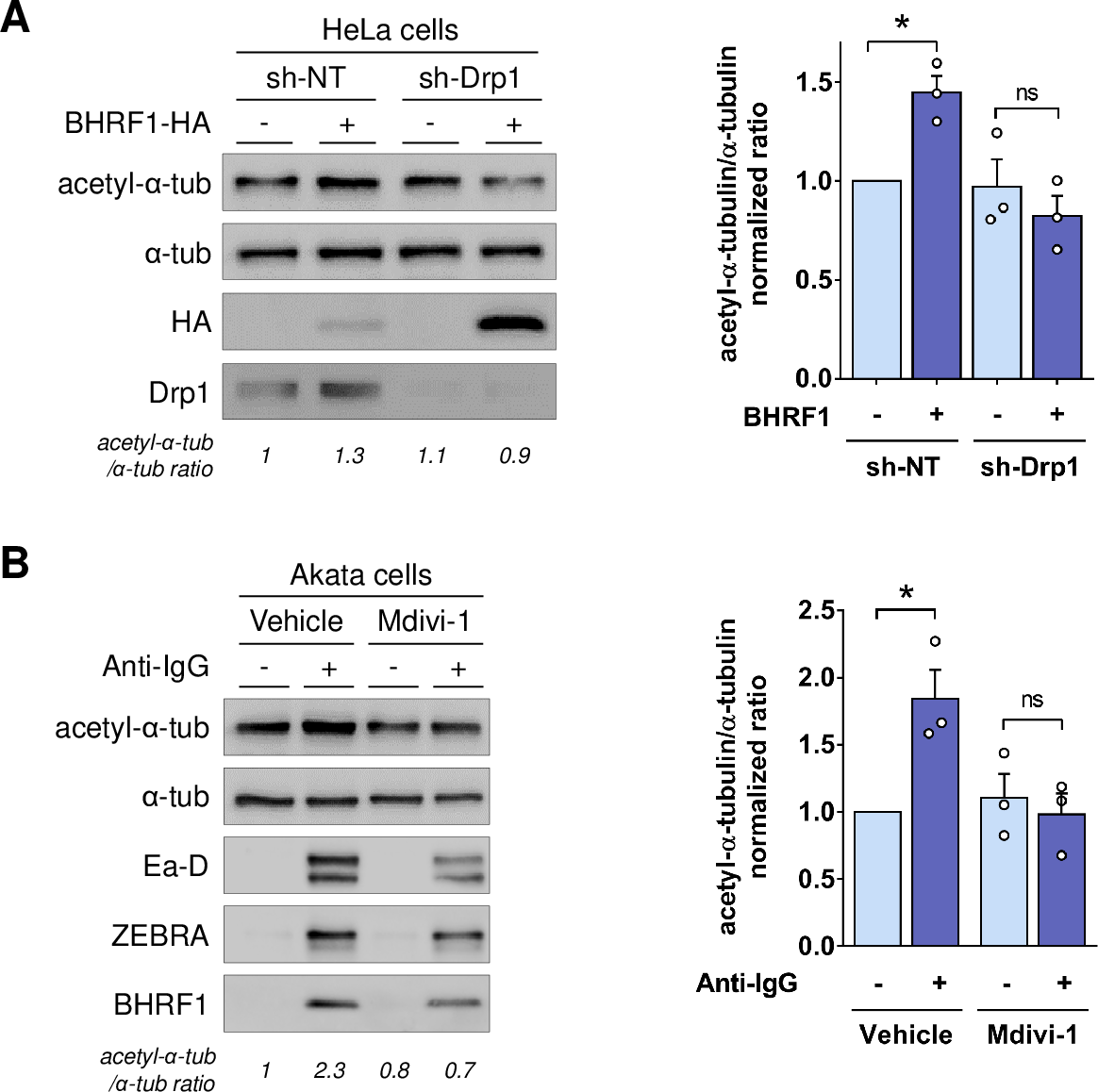

Supplement: S4 Fig — (A) HeLa cells expressing an sh-RNA against Drp1 (sh-Drp1) or a control sh-RNA (sh-NT) were transfected with BHRF1-HA for 24 h. Left, immunoblot analysis of acetyl-α-tubulin, α-tubulin, HA and Drp1. Right, normalized ratios of acetyl-α-tubulin to α-tubulin. (B) Akata cells are concomitantly treated for 24 h with anti-human IgG, to induce EBV reactivation, and with Mdivi-1, to inhibit Drp1. Left, immunoblot analysis of acetyl-α-tubulin, α-tubulin, Ea-D, ZEBRA and BHRF1. Right, normalized ratios of acetyl-α-tubulin to α-tubulin. Data represent the mean ± SEM of three independent experiments. ns = non-significant; * P < 0.05 (Student’s t-test). (TIF) [file ppat.1010371.s004.tif]

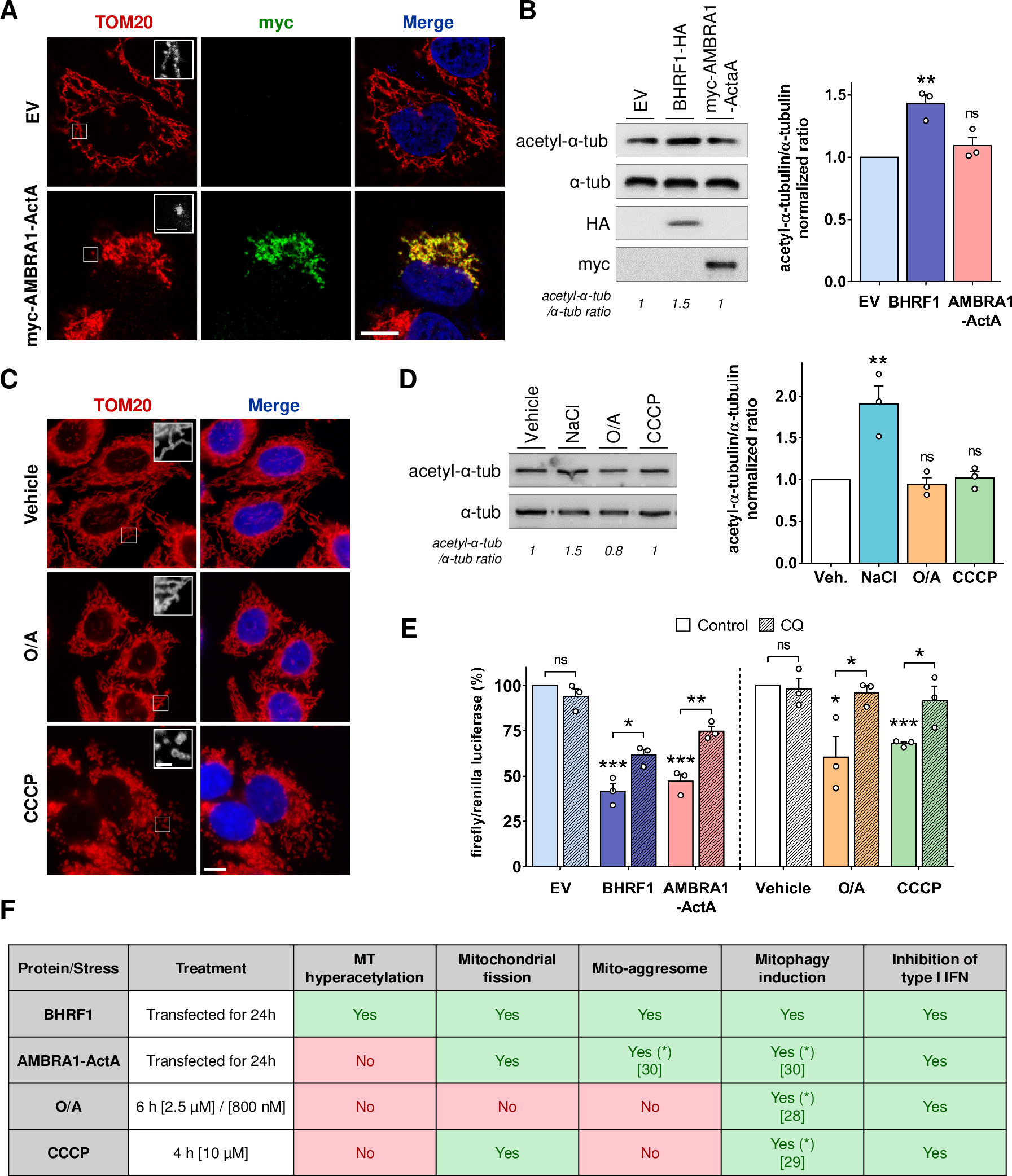

Supplement: S5 Fig — (A-B) HeLa cells were transfected with plasmids encoding myc-AMBRA1-ActA or BHRF1-HA for 24 h. (A) Confocal images with insets (3X) of AMBRA1- ActA-transfected cells immunostained for TOM20 and myc. Nuclei were stained with DAPI. Scale bars: 10 μm and 2 μm for insets. (B) Left, immunoblot analysis of acetyl-α-tubulin, α-tubulin, HA and myc. Right, normalized ratios of acetyl-α-tubulin to α-tubulin. (C-D) HeLa cells were treated for 6 h with O/A or 4 h with CCCP to induce mitophagy. (C) Representative images with insets (3X) of cells immunostained for TOM20. Nuclei were stained with DAPI. Scale bars: 10 μm and 2 μm for insets. (D) Left, immunoblot analysis of acetyl-α-tubulin and α-tubulin. Treatment with NaCl (30 min) was used as a positive control of hyperacetylation. Right, normalized ratios of acetyl-α-tubulin to α-tubulin. (E) Luciferase reporter assay on HEK293T cells treated with various mitophagy inducers. Cells are either transfected with BHRF1 or AMBRA1-ActA, or treated with O/A or CCCP. Before lysis, cells are treated or not with CQ for 4 h. Activation of the IFN-β promoter was analyzed 24 h post-transfection, or after indicated treatment. Firefly/renilla luciferase ratios were calculated and normalized to control conditions (EV or vehicle). (F) Summary table of the impact of mitophagy inducers on MT hyperacetylation and IFN inhibition. Results indicated by a star have been previously reported. Data represent the mean ± SEM of three independent experiments. ns = non-significant; * P < 0.05; ** P < 0.01; *** P < 0.001 (Student’s t-test). (TIF) [file ppat.1010371.s005.tif]

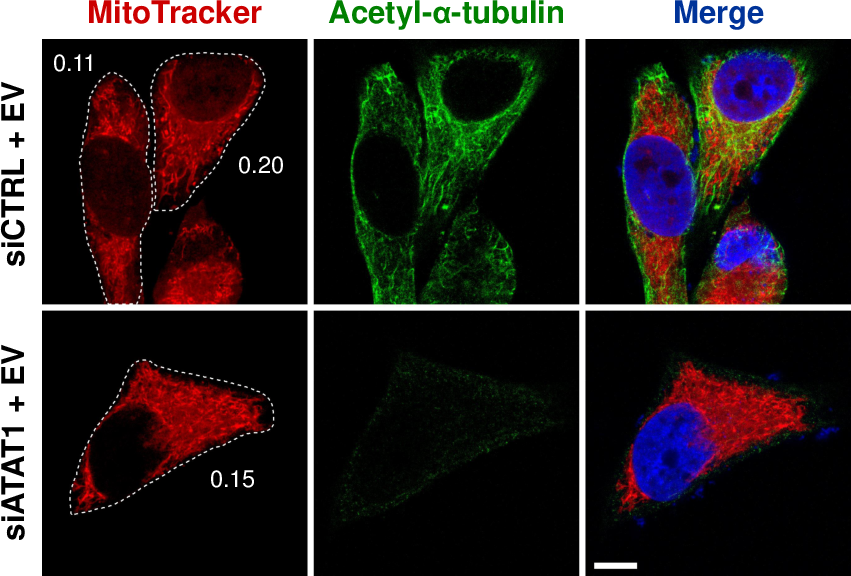

Supplement: S6 Fig — After knockdown of ATAT1, HeLa cells were transfected with an EV and fixed 24 h post-transfection. Mitochondria were labeled with MitoTracker, and cells were immunostained for acetyl-α-tubulin. Nuclei were stained with DAPI. Values of mitochondrial CI are indicated on representative cells. Scale bar: 20 μm. (TIF) [file ppat.1010371.s006.tif]

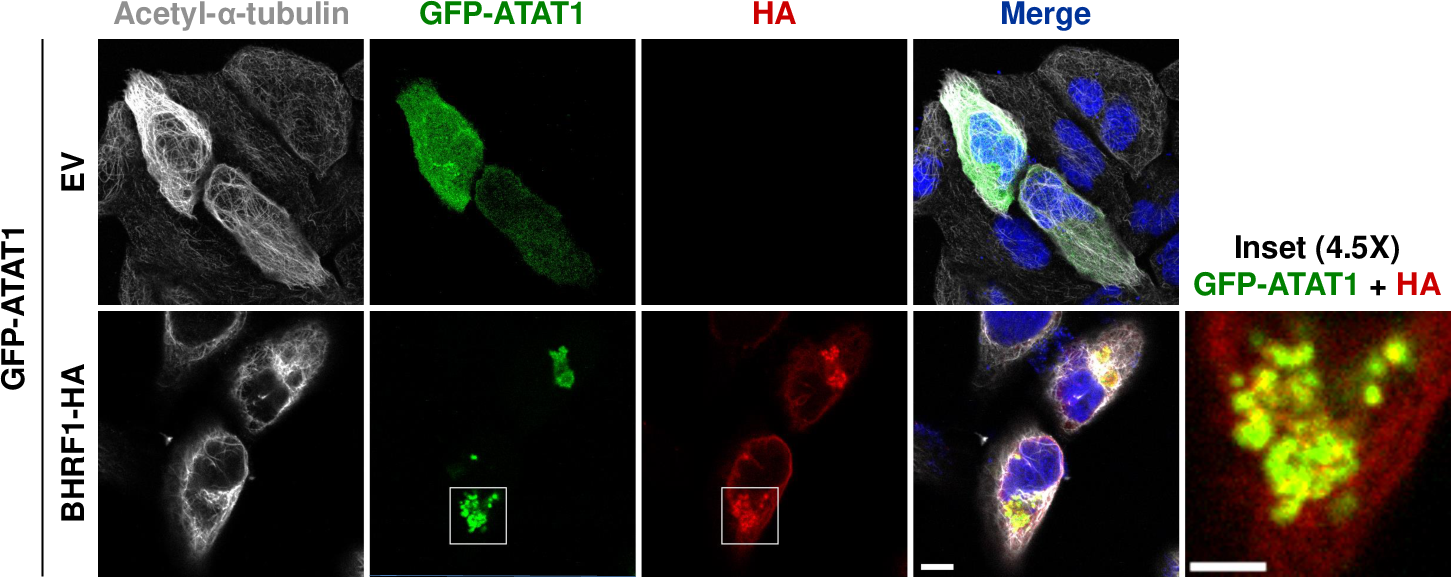

Supplement: S7 Fig — HeLa cells were co-transfected with GFP-ATAT1 and BHRF1-HA (or EV) for 24 h. Confocal images of cells immunostained for HA and acetyl-α-tubulin. Nuclei were stained with DAPI. Inset shows colocalization between ATAT1 and BHRF1. Scale bars: 10 μm and 5 μm for inset. (TIF) [file ppat.1010371.s007.tif]

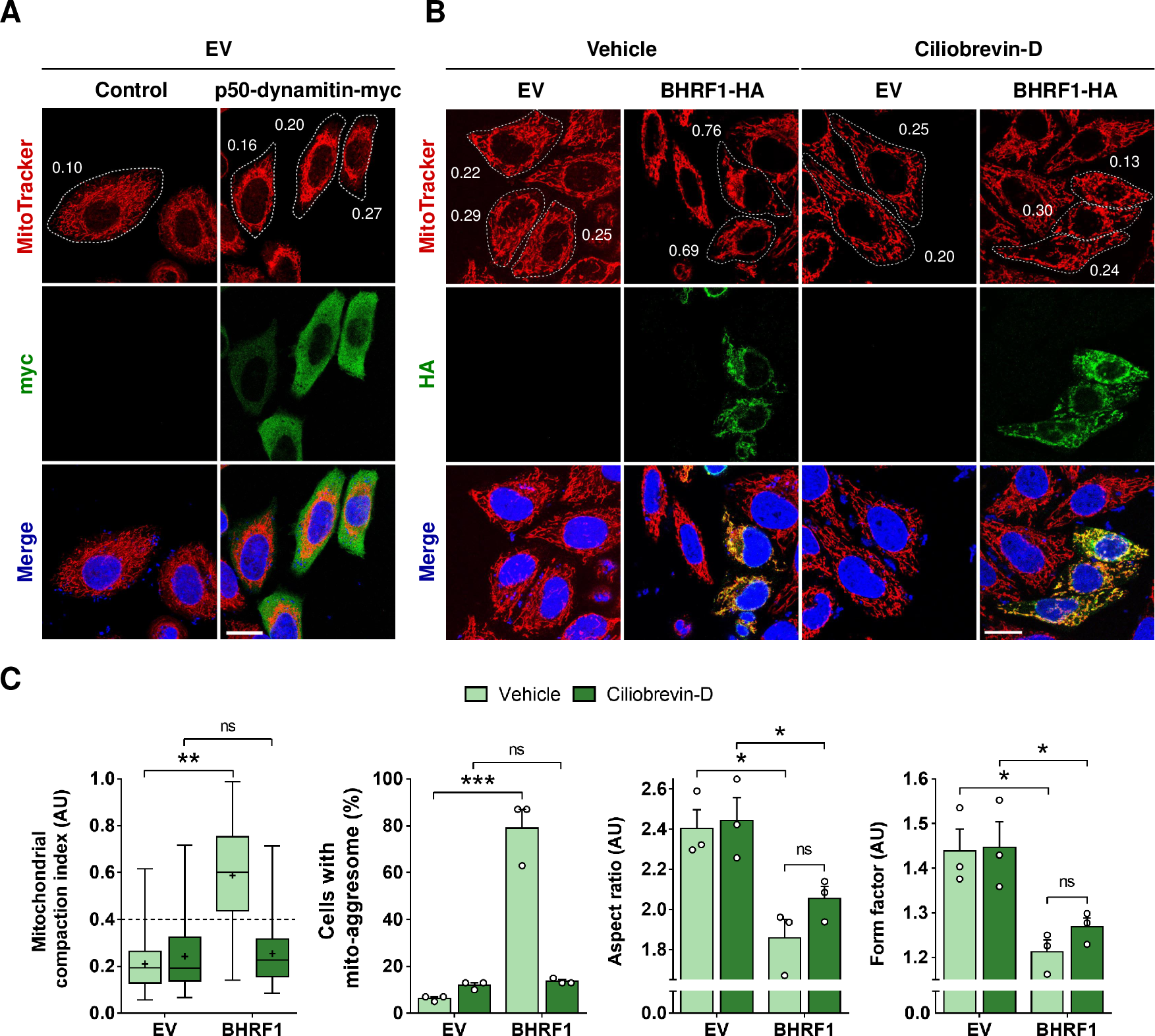

Supplement: S8 Fig — (A) Expression of p50-dynamitin-myc has no impact on the mitochondrial network. Confocal images of HeLa cells co-transfected with plasmids encoding EV and p50-dynamitin-myc for 24 h. Mitochondria were labeled with MitoTracker, and cells were immunostained for a myc-tag. Nuclei were stained with DAPI. Values of mitochondrial CI are indicated on representative cells. Scale bar: 20 μm. (B-C) HeLa cells were transfected for 24 h with BHRF1-HA (or EV) and treated overnight with ciliobrevin-D. (B) Confocal images. Mitochondria were labeled with MitoTracker, cells immunostained for HA and nuclei stained with DAPI. Values of mitochondrial CI are indicated on representative cells. Scale bar: 20 μm. (C) Quantification of CI, percentage of cells with a mitoaggresome and mitochondrial fission parameters (n = 20 cells per condition). Data represent the mean ± SEM of three independent experiments. ns = non-significant; * P < 0.05; ** P < 0.01; *** P < 0.001 (Student’s t-test). (TIF) [file ppat.1010371.s008.tif]

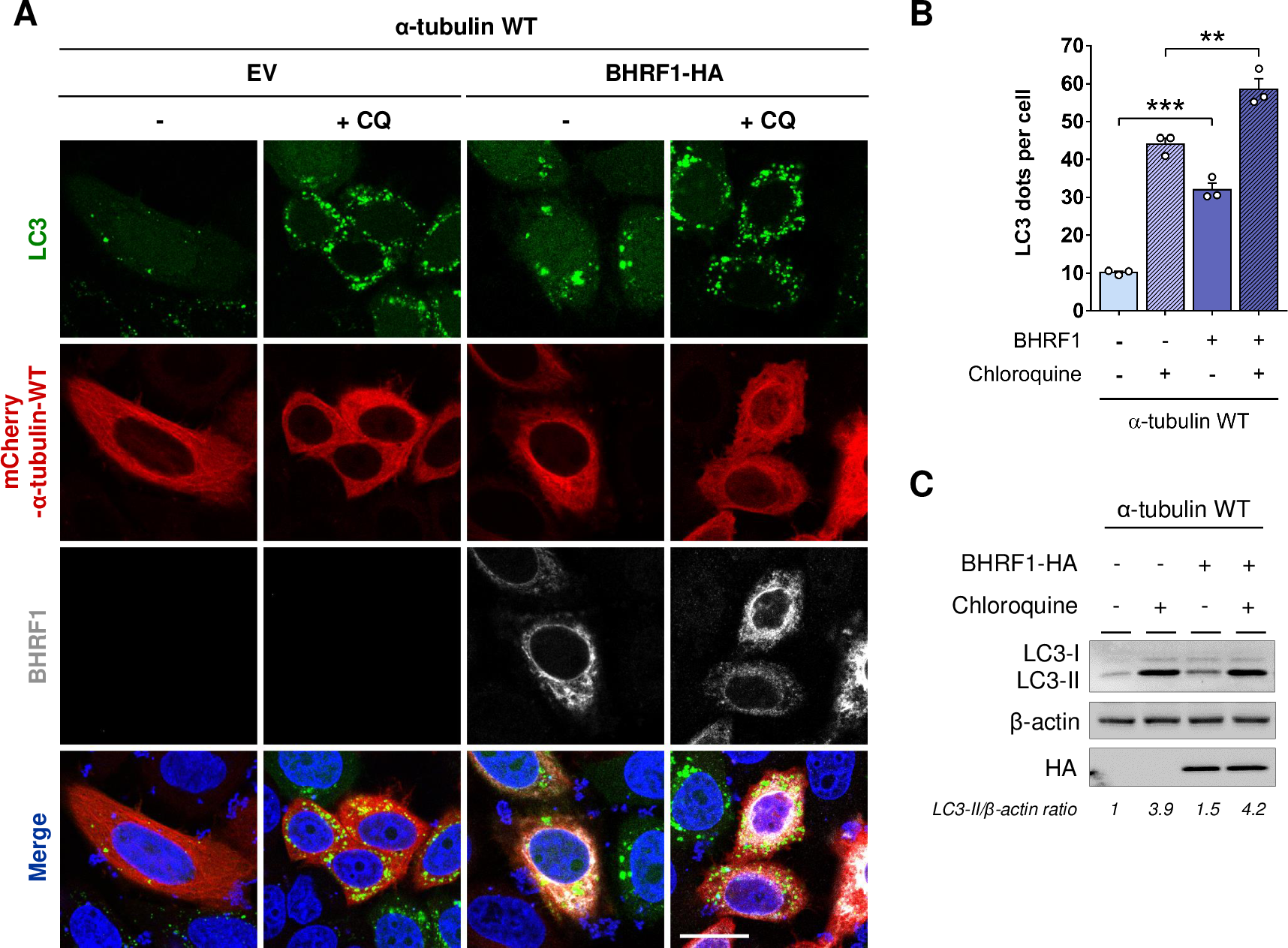

Supplement: S9 Fig — (A-C) HeLa cells co-transfected for 24 h with plasmids encoding BHRF1-HA (or EV) and mCherry-α-tubulin WT. Cells were treated with CQ when indicated. (A) Confocal images. Cells were immunostained for BHRF1 and LC3 and nuclei were stained with DAPI. Scale bar: 20 μm. (B) Quantification of LC3 dots (n = 30 cells per condition). (C) Immunoblot analysis of LC3 and BHRF1-HA expression. β-actin was used as a loading control. Data represent the mean ± SEM of three independent experiments. ** P < 0.01; *** P < 0.001 (Student’s t-test). (TIF) [file ppat.1010371.s009.tif]

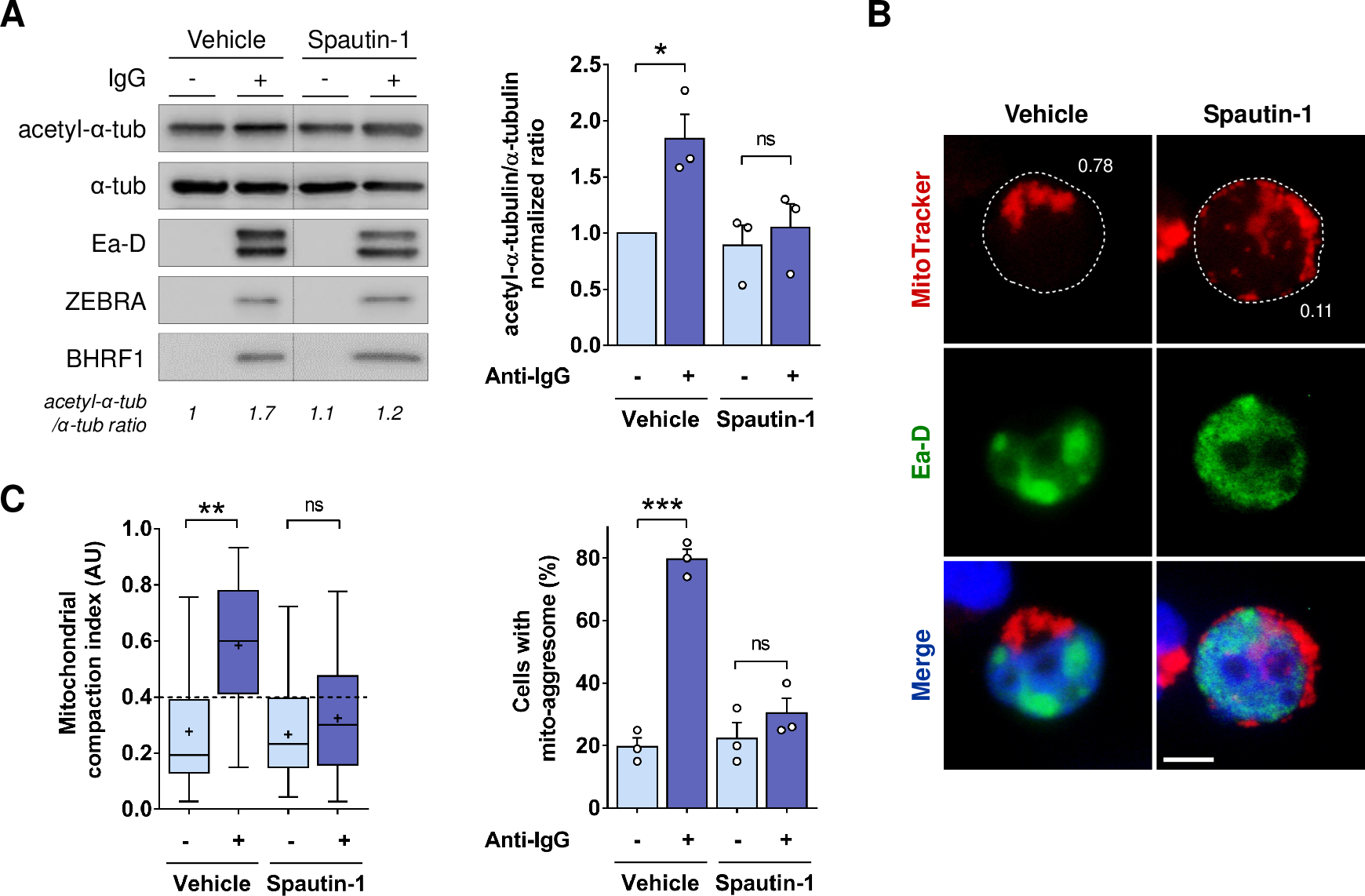

Supplement: S10 Fig — (A-C) EBV reactivation was induced in Akata cells by treatment with anti-human IgG for 24 h. At the same time, cells were treated with spautin-1 (or vehicle) for 24 h to block autophagy. (A) Left, immunoblot analysis of acetyl-α-tubulin, α-tubulin, Ea-D, ZEBRA and BHRF1. Right, normalized ratios of acetyl-α-tubulin to α-tubulin. (B) Representative images where mitochondria were labeled with MitoTracker, cells immunostained for EaD and nuclei stained with DAPI. Values of mitochondrial CI are indicated on each cell. Scale bar: 10 μm. (C) Quantification of CI and percentage of cells with a mito-aggresome (n = 20 cells per condition). Data represent the mean ± SEM of three independent experiments. ns = non-significant; * P < 0.05; ** P < 0.01; *** P < 0.001 (Student’s t-test). (TIF) [file ppat.1010371.s010.tif]
