# Supplementary material for: Essential role of hyperacetylated microtubules in innate immunity escape orchestrated by the EBV-encoded BHRF1 protein
Source: PLoS Pathog. 2022 Mar 11;18(3):e1010371. doi: 10.1371/journal.ppat.1010371 (PMC8942261; doi:10.1371/journal.ppat.1010371)
Supplement: Attachment — Submitted filename: 117991_1_rebuttal_2235736_r3v4yh.pdf [file ppat.1010371.s012.pdf]

Full Revision

Manuscript number: RC-2021-00890
Corresponding author: Audrey Esclatine

1. General Statements

We would like to thank the two reviewers for the valuable comments and suggestions on improvements. We addressed each reviewer's comments individually. We have carefully revised the manuscript to incorporate new data and to make necessary clarifications.

Overall we made the following major modifications:

1. We investigated the relevance of BHRF1 expression in the context of EBV infection, in B cells and epithelial cells. We observed that EBV reactivation leads to MT hyperacetylation and subsequent mito-aggresome formation in both cell types. An EBV^+ B cell line deficient for BHRF1 was generated and allowed us to demonstrate the involvement of BHRF1 in this phenotype. These results were added to Figures 2, 3 and Figure 1 – S1 in the revised version of the manuscript.

2. We better characterized the mechanism leading to MT hyperacetylation, by demonstrating that BHRF1 colocalizes and interacts with the tubulin acetyltransferase ATAT1. These results were added to Figure 5 and Figure 5 – S2 in the revised manuscript.

3. We generated stable HeLa cells KO for ATG5. Using these autophagy-deficient cells, we demonstrated the involvement of autophagy in BHRF1-induced MT hyperacetylation and mito-aggresome formation. We added these results to Figure 8 in the revised version of the manuscript.

4. We compared the impact of BHRF1 with other mitophagy inducers on MT hyperacetylation, mitochondrial morphodynamics and the inhibition of IFN production, to demonstrate the specificity of the mechanism of action of BHRF1 (Figure 4 – S1).

5. We demonstrated that MT hyperacetylation requires mitochondrial fission, using a Drp1-deficient HeLa cell line that we have previously described (Vilmen *et al.*, 2020). This result was added to the revised version of the manuscript in Figure 3 – S2A. Moreover, we confirmed this result in the context of EBV infection (Figure 3 – S2B).

2. Point-by-point description of the revisions

Reviewer #1 (Evidence, reproducibility and clarity)

Major comments:

1. In the presented manuscript the authors characterize mainly BHRF1 overexpression in HeLa cells. Does BHRF1 also block type I IFN responses by microtubule hyperacetylation in the context of EBV infection? Do alpha-tubulin K40A overexpressing B cells produce more type I IFN after EBV infection?

In the revised version of the manuscript, we added several experiments to explore the phenotype of BHRF1 during EBV infection, as requested by the two reviewers. Since EBV infects both B cells and epithelial cells, we used two different approaches. In latently-infected B cells, coming from Burkitt lymphoma (Akata cells), we induced EBV reactivation by anti-IgG treatment. To explore the importance of BHRF1 in this cell type, we constructed a cell line knocked down for BHRF1 expression, thanks to a lentivirus bearing an shRNA against BHRF1. In parallel, HEK293 cells harboring either EBV WT or EBV ΔBHRF1 genome were transfected with ZEBRA and Rta plasmids to induce the viral productive cycle in epithelial cells.

We demonstrated that EBV infection induces MT hyperacetylation and subsequent mito-aggresome formation, both dependent on autophagy. Moreover, this phenotype requires BHRF1 expression in B cells and epithelial cells. We also observed that the expression of alpha-tubulin K40A in EBV^+ epithelial cells blocks mito-aggresome formation induced by EBV reactivation. These results are now presented in Figures 2 and 3 in the revised version of the manuscript.

Regarding regulation of IFN response during infection, several EBV-encoded proteins and non-coding RNAs have been described to interfere with the innate immune system. For example, BGLF4 and ZEBRA bind to IRF3 and IRF7, respectively, to block their nuclear activity (Hahn et al., 2005; Wang et al., 2009). Moreover, Rta expression decreases mRNA expression of IRF3 and IRF7 (Bentz et al., 2010; Zhu et al., 2014). We therefore think that studying the inhibitory role of BHRF1 on IFN response in the context of EBV reactivation will be arduous. Indeed, the lack of BHRF1 could be compensated by the activity of other viral proteins acting on innate immunity.

2. The authors document that the observed microtubule hyperacetylation is due to the acetyltransferase ATAT1. How does BHRF1 activate ATAT1? Is there any direct interaction?

As requested by reviewer#1, we explored a possible interaction of BHRF1 and ATAT1. First, we observed by confocal microscopy that GFP-ATAT1 colocalized with BHRF1 in the

juxtanuclear region of HeLa cells (Figure 5 – S2). Second, we demonstrated by two co-immunoprecipitation assays that BHRF1 binds to exogenous ATAT1 (Figures 5E and 5F). These new results have been added to the revised version of the manuscript and clarify the mechanism of action of BHRF1.

To go further, we explored whether BHRF1 was able to stabilize ATAT1 because it was recently reported that p27, an autophagy inducer that modulates MT acetylation, binds to and stabilizes ATAT1 (Nowosad et al., 2021). However, BHRF1 expression does not impact the expression of ATAT1 (data not shown).

3. Furthermore, the authors demonstrate with pharmacological autophagy inhibitors that autophagy is increased in a BHRF1 dependent and microtubule acetylation independent manner but required for microtubule hyperacetylation. How does autophagy stimulate ATAT1 dependent microtubule hyperacetylation? Is this dependency also observed with a more specific ATG silencing or knock-out?

We generated a stable autophagy-deficient HeLa cell line KO for ATG5, using an ATG5 CRISPR/Cas9 construct delivered by a lentivirus. The lack of ATG5 expression and LC3 lipidation was verified by immunoblot (Figure 8B). We observed that BHRF1 was unable to increase MT acetylation in this autophagy-deficient cell line (Figure 8C) in accordance with our data reported in the original manuscript using treatment with spautin 1 or 3-MA (previously Figure S5C and Figure 8A in the revised version). Moreover, the lack of hyperacetylated MT in BHRF1-expressing cells led to a dramatic reduction of mito-aggresome formation (Figures 8D and 8E). These new results demonstrate that autophagy is required for BHRF1-induced MT hyperacetylation.

Minor comments:

1. "Innate immunity" and "innate immune system", but not "innate immunity system" are in my opinion better wordings.

We thank reviewer #1 for this useful comment. The term “innate immunity system” in the introduction section has been replaced by “innate immune system”. Elsewhere, we used “innate immunity”.

2. The reader would benefit from a discussion on the role of type I IFNs during EBV infection and how important the authors think their new mechanism could be in this context.

We thank the reviewer for this suggestion. However, we already discussed the different strategies developed by EBV to counteract IFN response induction, in our previous study,

suggesting the importance of IFN in the control of EBV infection (Vilmen *et al.*, 2020). In this study, we have focused the discussion on the role of mitophagy in the control of IFN production.

Reviewer #1 (Significance):

The significance of the described pathway for type I IFN production needs to be documented in the context of EBV infection.

The revised version of the manuscript now explored the role of BHRF1 in the context of EBV infection See above for details (major comment 1).

Reviewer #2 (Evidence, reproducibility and clarity)

The work presented is a relatively straightforward cell biological dissection of a subset of the previously described functions of BHRF1, focusing on the mitochondrial aggregation phenotype. The approaches and analysis are performed in cell lines mainly using overexpression and some siRNA experiments and appear well done throughout.

We thank reviewer #2 for this comment and would like to underline that the revised version of the manuscript includes now a study of BHRF1 in the context of infection in both B cells and epithelial cells, the generation of a stable EBV positive B cells KD for BHRF1 by using shRNA approach and the generation of a stable autophagy-deficient cell line, using CRISPR/cas9 against ATG5.

Reviewer #2 (Significance):

The current study unpicks one of the phenotypes induced by BHRF1 over expression: namely the previously reported mitochondrial aggregation phenotype. The findings that peri-nuclear mitochondrial aggregation are dependent on microtubules and retrograde motors are useful but could perhaps have been predicted. Overexpression of many proteins (or indeed chemical treatments) causing cellular and / or mitochondrial stress have been shown to cause mitochondrial perinuclear aggregation.

To explore the specificity of BHRF1 activity on mito-aggresome formation, we decided to investigate the impact of AMBRA1-ActA, a previously characterized mitophagy inducer, on MT (Strappazzon et al., 2015). We observed that expression of AMBRA1-ActA leads to mito-aggresome formation but does not modulate acetylation of MTs, contrary to BHRF1. This result was added to the revised version of the manuscript (Figure 4 - S1A and S1B). Moreover, chemical

treatments with either oligomycin/antimycin or CCCP, which induce mitochondrial stress and mitophagy (Lazarou et al., 2015; Narendra et al., 2008), do not cause mitochondrial juxtanuclear aggregation (Figure 4 - S1C). We also observed that a hyperosmotic shock-induced by NaCl leads to MT hyperacetylation (Figure 4 - S1D) but not to the mito-aggresome formation (data not shown), suggesting that MT hyperacetylation *per se* is not sufficient to induce the clustering of mitochondria. Altogether, these new results demonstrated the originality of the mechanism used by BHRF1 to induce mito-aggresome formation.

The findings linking the process to altered tubulin acetylation are more novel and interesting and may add a new dimension to understanding of BHRF1 function. However what is lacking here is really advancing our understanding of how BHRF1 does this.

We thank the reviewer for underlining the fact that regulation of mitochondrial morphodynamics by BHRF1 via MT hyperacetylation is novel and interesting.

In the original version of the manuscript, we have demonstrated that autophagy and ATAT1 are required for BHRF1-induced hyperacetylation. In the revised version, we uncovered that BHRF1 interacts and colocalizes with ATAT1 (Figures 5E, 5F and Figure 5 – S2). Moreover, we demonstrated that MT hyperacetylation is involved in the localization of autophagosomes next to the nucleus, thus close to the mito-aggresome. Therefore, we better characterized the mechanism of action of BHRF1 in the revised manuscript.

Although some downstream processes are identified in the current and previous study it still remains unclear what the exact underlying mechanisms are. Is BHRF1 doing this by disrupting mitochondrial function and making the organelles sick or by causing cellular stress indirectly leading to mitochondrial pathology? Previous studies have shown that cellular stress such as altered proteostasis can also cause stress-induced mitochondrial retrograde trafficking and aggregation. Is BHRF1 causing the same phenotype by generally stressing the cell and if it is more specifically through mitochondrial disruption what is the mechanism? As demonstrated by the authors in their previous work, BHRF1 does a number of things to cell signalling. Which of these are leading to a general disruption of cell signalling versus having specific effects on the cell or mitochondria still seems somewhat unclear.

We previously reported that BHRF1 expression does not alter the mitochondrial membrane potential (Vilmen *et al.*, 2020). contrary to treatment by O/A or CCCP. Moreover, we observed that these treatments do not induce mitochondrial clustering (Figure 4 – S1). Therefore, BHRF1 modulates mitochondrial dynamics in a specific and regulated manner.

Our study clearly demonstrated that BHRF1 uses an original strategy to modulate IFN response, via a regulated pathway of successive steps, from mitochondrial fission to mitophagy, via MT hyperacetylation, rather than “a general disruption of cell signalling”.

It would be interesting to know whether the role of microtubule hyperacetylation and ATAT1 are more generally involved in other previously described processes of stress induced mitochondrial aggregation.

In the revised version of the manuscript, we observed that AMBRA1-ActA does not change the level of MT acetylation, whereas it induces mito-aggresome formation. These data reinforce the originality of the BHRF1 mechanism.

Currently while this is a nicely performed follow up study to their 2020 paper, the present study neither provides in depth mechanistic advance of BHRF1 function, nor a better understanding of the molecular steps in a more generally relevant pathway (e.g. mitophagy).

We disagree with the reviewer’s comment. Indeed, in this new study, we uncovered and characterized a new mechanism of action for BHRF1 via ATAT1-dependent MT hyperacetylation. More generally, we reported for the first time that innate immunity can be regulated by the level of MT acetylation.

In addition, all the experiments were performed in cell lines and rely on the overexpression of a viral protein. But this is a significant over-simplification of the viral pathological process. It therefore remains unclear how pathophysiologically relevant the findings are (e.g. to EBV pathology) without further extending this element of the work.

To address this comment, we extended our results in the infectious context, by adding several experiments performed in EBV-infected cell lines (see above reviewer#1 for details). The same phenotype was observed after reactivation of the EBV productive cycle as in BHRF1 ectopic expression. Moreover, we demonstrated that the phenotype is BHRF1-dependent. This suggests the importance of BHRF1 in EBV pathogenesis by participating in innate immunity control.

An additional minor issue is the authors naming of the process as Mito-aggresome formation. Although this might sound catchy it is somewhat unclear what the biological basis for this is. Aggresomes are defined structures that occur in cells during pathology and due to the peri-nuclear accumulation of misfolded protein. Since the process here is simply the description of aggregated mitochondria next to the nucleus but doesn't seem to have anything to do with protein misfolding it's really unclear how this labelling is helpful to the field. The process of perinuclear mitochondrial aggregation e.g. during mitochondrial stress or damage has been

described many times before without the need for calling it a mito-aggresome. This term is likely to cause unhelpful confusion.

We understand the comment of reviewer #2, but since 2010 the term “mito-aggresome” was previously used in other studies and refers to a clustering of mitochondria next to the nucleus, similarly to what we observed with BHRF1 (D’Acunzo *et al.*, 2019; Lee *et al.*, 2010; Springer and Kahle, 2011, 2011; Strappazzon *et al.*, 2015; Van Humbeeck *et al.*, 2011; Yang and Yang, 2011).

However, we took into consideration the risk of confusion for the readers, by changing how we introduced the term “mito-aggresome” in the revised version of the manuscript (page 5 line 94).

3. References

Bentz GL, Liu R, Hahn AM, Shackelford J, Pagano JS. 2010. Epstein–Barr virus BRLF1 inhibits transcription of IRF3 and IRF7 and suppresses induction of interferon-β. *Virology* **402**:121–128. doi:10.1016/j.virol.2010.03.014

D’Acunzo P, Strappazzon F, Caruana I, Meneghetti G, Di Rita A, Simula L, Weber G, Del Bufalo F, Dalla Valle L, Campello S, Locatelli F, Cecconi F. 2019. Reversible induction of mitophagy by an optogenetic bimodular system. *Nat Commun* **10**:1533. doi:10.1038/s41467-019-09487-1

Hahn AM, Huye LE, Ning S, Webster-Cyriaque J, Pagano JS. 2005. Interferon regulatory factor 7 is negatively regulated by the Epstein-Barr virus immediate-early gene, BZLF-1. *J Virol* **79**:10040–10052. doi:10.1128/JVI.79.15.10040-10052.2005

Lazarou M, Sliter DA, Kane LA, Sarraf SA, Wang C, Burman JL, Sideris DP, Fogel AI, Youle RJ. 2015. The ubiquitin kinase PINK1 recruits autophagy receptors to induce mitophagy. *Nature* **524**:309–314. doi:10.1038/nature14893

Lee J-Y, Nagano Y, Taylor JP, Lim KL, Yao T-P. 2010. Disease-causing mutations in Parkin impair mitochondrial ubiquitination, aggregation, and HDAC6-dependent mitophagy. *J Cell Biol* **189**:671–679. doi:10.1083/jcb.201001039

Narendra DP, Tanaka A, Suen D-F, Youle RJ. 2008. Parkin is recruited selectively to impaired mitochondria and promotes their autophagy. *J Cell Biol* **183**:795–803. doi:10.1083/jcb.200809125

Nowosad A, Creff J, Jeannot P, Culerrier R, Codogno P, Manenti S, Nguyen L, Besson A. 2021. p27 controls autophagic vesicle trafficking in glucose-deprived cells via the regulation of ATAT1-mediated microtubule acetylation. *Cell Death Dis* **12**:1–18. doi:10.1038/s41419-021-03759-9

Springer W, Kahle PJ. 2011. Regulation of PINK1-Parkin-mediated mitophagy. *Autophagy* **7**:266–278. doi:10.4161/auto.7.3.14348

Strappazzon F, Nazio F, Corrado M, Cianfanelli V, Romagnoli A, Fimia GM, Campello S, Nardacci R, Piacentini M, Campanella M, Cecconi F. 2015. AMBRA1 is able to induce mitophagy via

Review COMMONS

LC3 binding, regardless of PARKIN and p62/SQSTM1. *Cell Death Differ* **22**:419–32. doi:10.1038/cdd.2014.139

Van Humbeeck C, Cornelissen T, Hofkens H, Mandemakers W, Gevaert K, De Strooper B, Vandenberghe W. 2011. Parkin Interacts with Ambra1 to Induce Mitophagy. *J Neurosci* **31**:10249–10261. doi:10.1523/JNEUROSCI.1917-11.2011

Vilmen G, Glon D, Siracusano G, Lussignol M, Shao Z, Hernandez E, Perdiz D, Quignon F, Mouna L, Poüs C, Gruffat H, Maréchal V, Esclatine A. 2020. BHRF1, a BCL2 viral homolog, disturbs mitochondrial dynamics and stimulates mitophagy to dampen type I IFN induction. *Autophagy* **17**:1296–1315. doi:10.1080/15548627.2020.1758416

Wang J-T, Doong S-L, Teng S-C, Lee C-P, Tsai C-H, Chen M-R. 2009. Epstein-Barr Virus BGLF4 Kinase Suppresses the Interferon Regulatory Factor 3 Signaling Pathway. *J Virol* **83**:1856–1869. doi:10.1128/JVI.01099-08

Yang J-Y, Yang WY. 2011. Spatiotemporally controlled initiation of Parkin-mediated mitophagy within single cells. *Autophagy* **7**:1230–1238. doi:10.4161/auto.7.10.16626

Zhu L-H, Gao S, Jin R, Zhuang L-L, Jiang L, Qiu L-Z, Xu H-G, Zhou G-P. 2014. Repression of interferon regulatory factor 3 by the Epstein-Barr virus immediate-early protein Rta is mediated through E2F1 in HeLa cells. *Mol Med Rep* **9**:1453–1459. doi:10.3892/mmr.2014.1957
